# Overgrowth of long bone in rabbits by growth stimulation through metaphyseal hole creation

**DOI:** 10.1038/s41598-023-36278-y

**Published:** 2023-06-07

**Authors:** Kyoung-Mi Lee, Eun Ae Ko, Mudit Shah, Seung Eun Oh, Jin Woo Lee, Byoung Kyu Park, Hyun Woo Kim, Kun-Bo Park

**Affiliations:** 1 Department of Orthopaedic Surgery, Yonsei University College of Medicine, Seoul, 03722 Republic of Korea; 2 Brain Korea 21 Plus Project for Medical Science, Yonsei University College of Medicine, Seoul, 03722 Republic of Korea; 3 Department of Orthopaedic Surgery, Inje University Haeundae Paik Hospital, Busan, Republic of Korea; 4 Division of Pediatric Orthopedic Surgery, Severance Children’s Hospital, Yonsei University College of Medicine, 50-1 Yonsei-Ro, Seodaemun-Gu, Seoul, 03722 Republic of Korea

**Keywords:** Medical research, Risk factors

## Abstract

Overgrowth of long bones was noted in pediatric patients who underwent anterior cruciate ligament reconstruction. Hyperaemia during creating a metaphyseal hole and the microinstability made by the drill hole may induce overgrowth. This study aimed to determine whether metaphyseal hole creation accelerates growth and increases bone length and compare the effects of growth stimulation between metaphyseal hole creation and periosteal resection. We selected 7- to 8-week-old male New Zealand white rabbits. Periosteal resection (N = 7) and metaphyseal hole creation (N = 7) were performed on the tibiae of skeletally immature rabbits. Seven additional sham controls were included as age-matched controls. In the metaphyseal hole group, the hole was made using a Steinman pin at the same level of periosteal resection, and the cancellous bone beneath the physis was removed by curettage. The vacant space in the metaphysis below the physis was filled with bone wax. Tibiae were collected 6 weeks after surgery. The operated tibia was longer in the metaphyseal hole group (10.43 ± 0.29 cm vs. 10.65 ± 0.35 cm, *P* = 0.002). Overgrowth was higher in the metaphyseal hole group (3.17 ± 1.16 mm) than in the sham group (− 0.17 ± 0.39 mm, *P* < 0.001). The overgrowth in the metaphyseal hole group was comparable to that in the periosteal resection group (2.23 ± 1.52 mm, *P* = 0.287). In rabbits, metaphyseal hole creation and interposition with bone wax can stimulate long bone overgrowth, and the amount of overgrowth is similar to that seen in periosteal resection.

## Introduction

Overgrowth of long bones has been reported in paediatric populations^1–4^. Due to overgrowth after fracture, growth stimulation by periosteal stripping or division has been mentioned as one of the causes, and the effect of circumferential periosteal damage on overgrowth has been proven through diverse animal studies^5–8^. However, the overgrowth of a long bone is also noted without wide periosteal damage. Overgrowth was also noted in pediatric patients who had undergone anterior cruciate ligament (ACL) reconstruction^3,9^. During ACL reconstruction, there was no circumferential periosteal damage; instead, the drill hole was made from the metaphysis to the epiphysis, and the hole was filled with a tendon graft. The overgrowth was also reported in children with proximal metaphyseal fracture as Cozen’s phenomenon, and the overgrowth after proximal cortical breakage without extensive periosteal damage was confirmed in an animal study^10,11^. It is possible that, in addition to periosteal damage, there may be another mechanism related to the overgrowth of the bone.

Increased metabolic activity at the physis or bony instability has been suggested to the other possible cause of the overgrowth^12,13^. The cortical hole made by drilling at the metaphysis could increases metabolic activity at the physis through hyperaemia and the large cortical hole would be related to the bony instability. During ACL reconstruction, a cortical hole made at the metaphysis to the phyis and interposition of the vacant space with the tendon may promote hyperaemia and bony microinstability^3,14^.

We hypothesized that creating a metaphyseal hole and interposition with material different from the bone, such as tendon, may induce overgrowth. This study aimed to determine whether metaphyseal hole creation accelerates bony growth and increases the bone length and compare the effects of metaphyseal hole creation on growth stimulation with periosteal resection.

## Results

### Overgrowth of tibia after the creation of metaphyseal hole

**Figure 1:**
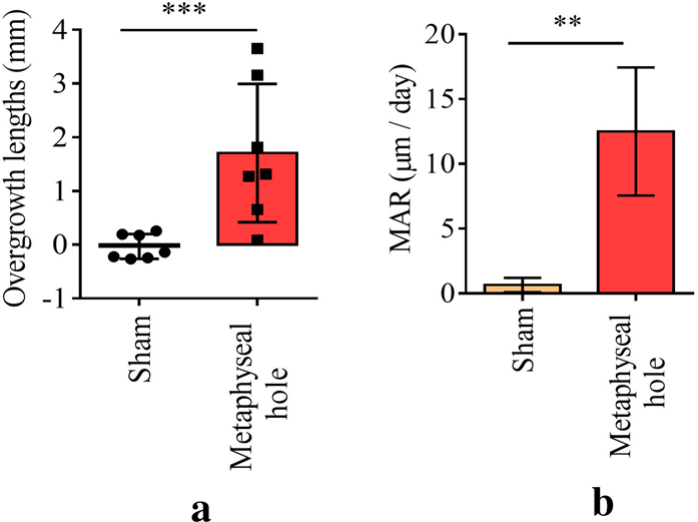
Overgrowth of rabbit tibiae in sham and metaphyseal hole groups. (**a**) Quantification analysis of the overgrowth lengths of sham and metaphyseal hole groups using radiographic images of rabbit tibiae (n = 7 per group). Overgrowth lengths were measured by Image J software. (**b**) Quantification analysis of mineral apposition rate (MAR) sham and metaphyseal hole groups using the double calcein labeling images (n = 3 per group). MAR (µm/day) = double fluorescence line spacing/days between the two lines. *P < 0.05, **P < 0.01, ***P < 0.001 compared to sham.

In the sham control group, there was no difference in tibia length between the control (107.1 ± 2.3 mm) and operation side (106.7 ± 2.3 mm, *P* = 0.166). However, in the metaphyseal hole group, the operation side was longer (104.3 ± 2.9 mm vs. 106.5 ± 3.5 mm, *P* = 0.002). The average overgrowth was 3.17 ± 1.16 mm in the metaphyseal hole group (*P* < 0.001) compared with -0.17 ± 0.39 mm in the sham group (Fig. 1a).

In H&E staining, the height of the growth plate was more significant in the metaphyseal hole group (273.1 ± 15.9 µm) compared to the sham group (206.6 ± 13.4 µm) (*P* < 0.001). The growth rate in calcein bone labelling was also higher in the metaphyseal hole group (12.5 ± 4.94 µm/day) compared to the sham group (0.66 ± 0.55 µm/day) (*P* < 0.01) (Fig. 1b).

### Comparison of growth stimulation between metaphyseal hole creation and periosteal resection

**Figure 2:**
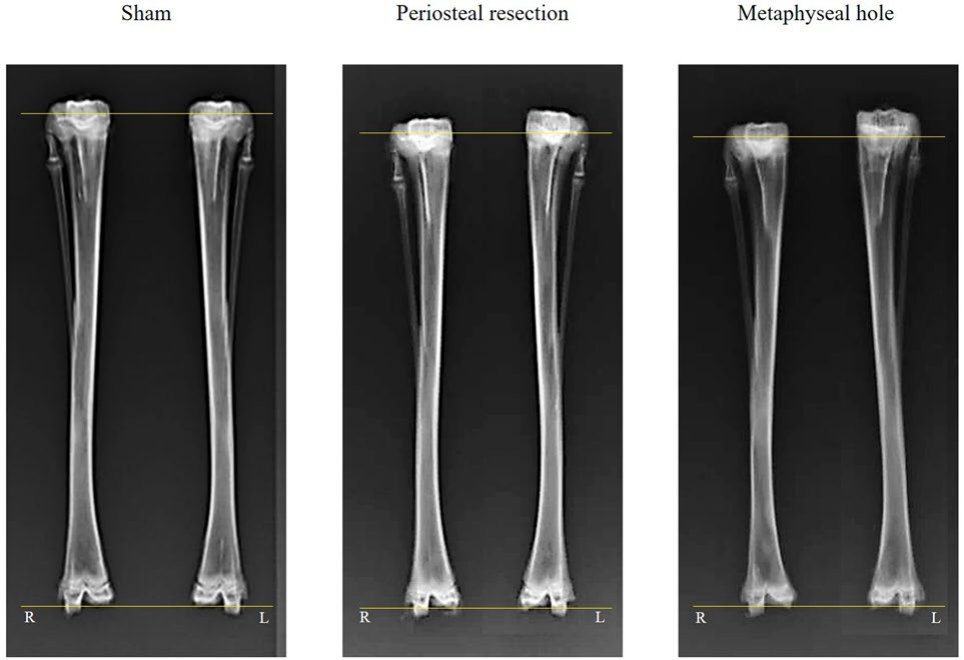
Radiographic images of rabbit tibiae at 6 weeks after surgery. Surgery was performed on the left tibiae.

In the periosteal resection group, the operation side was longer than the non-operated side (107.5 ± 2.0 mm vs. 105.3 ± 2.2 mm, *P* = 0.008), and the overgrowth (2.23 ± 1.52 mm) was significantly higher compared to the sham group (− 0.17 ± 0.39 mm, *P* < 0.001). However, there was no difference in the overgrowth length between the periosteal resection and metaphyseal hole groups (*P* = 0.219) (Fig. 2).

**Figure 3:**
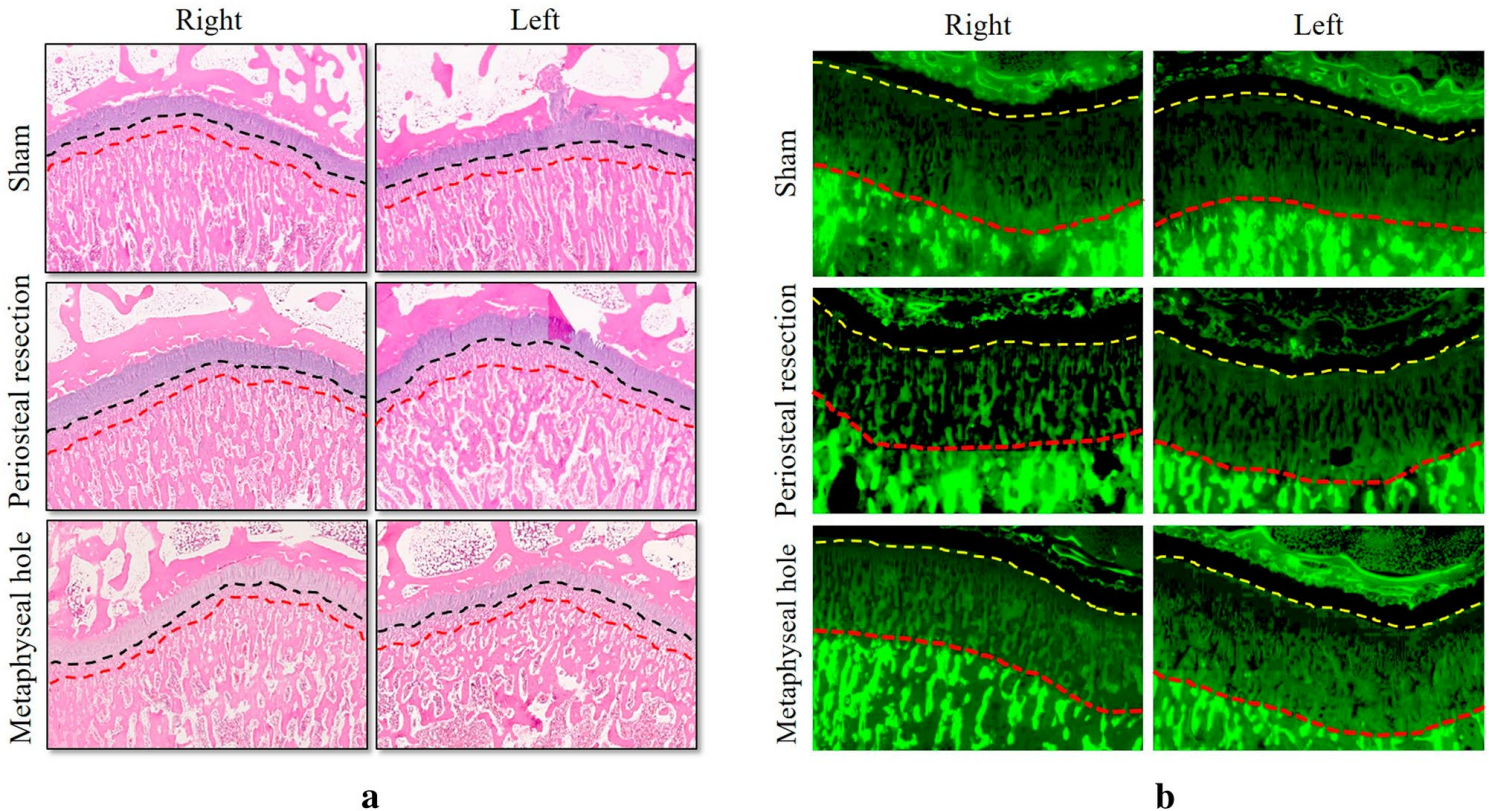
Histological analysis of bone growth after surgery. (**a**) Haematoxylin and eosin staining for overgrowth bone using decalcified bone tissues. The distance between red and black dotted lines represents a new bone area scale bar, 2 mm. (**b**) Bone dynamic histomorphometry analysis for assessment of growth rate on tibia using un-decalcified bone specimens. For dynamic histomorphometric analysis, calcein (15 mg/kg) was subcutaneously injected into the rabbits 3 and 7 days before sacrifice. Calcein double labels were measured using fluorescence microscopy. The distance between the red and yellow dotted lines represents a new bone area.

In the periosteal resection group, the height of the growth plate (258.0 ± 14.1 µm) and the growth rate (11.73 ± 2.51 µm/day) were higher than those in the sham group (*P* = 0.017, *P* < 0.05, respectively) (Fig. 3). However, there was no difference in the height of the growth plate and growth rate (*P* = 0.287 and *P* = 0.661, respectively) between the periosteal resection and metaphyseal hole groups (Table 1).

**Table 1:** Comparisons of overgrowth, growth plate height, and growth rate.

	Metaphyseal hole	Periosteal resection	Sham	*P*-value		
				Metaphyseal hole *vs.* Sham	Periosteal resection *vs.* Sham	Metaphyseal hole *vs.* Periosteal resection
Overgrowth of tibia (mm)	3.17 ± 1.16	2.23 ± 1.52	− 0.17 ± 0.39	< 0.001	< 0.001	0.219
Height of growth plate (µm)	273.1 ± 15.9	258.0 ± 14.1	206.6 ± 13.4	< 0.001	0.017	0.287
Growth rate (µm/day)	12.5 ± 4.94	11.73 ± 2.51	0.66 ± 0.55	0.009	0.001	0.661

## Discussion

Overgrowth of long bones is encountered in the pediatric population. Femoral overgrowth after fracture is a well-known phenomenon, and periosteum detachment or injury has been thought to be one of the causes of overgrowth. However, overgrowth has also been observed after ACL reconstruction. In these procedures, there is not a periosteal detachment or division. Instead, the cortical bone penetration, removal of cancellous bone, and filling the vacant space with tendon are the key components. We hypothesized that the creation of a metaphyseal hole and interposition with other material from the bone might stimulate overgrowth. The tibia of rabbits demonstrated a significant overgrowth after metaphyseal hole creation and interpositioning with bone wax wherein the amount of overgrowth was comparable to that of the periosteal procedure.

The length of the bone in the metaphyseal hole group was significantly greater than that on the opposite side. The overgrowth in the metaphyseal hole group was 3.17 ± 1.16 mm, which was approximately 2.9% of the length of the operated limb. Cohen et al*.* reviewed 26 cases of transphyseal ACL reconstructions^14^. They reported an average 1.2 ± 3.2 mm overgrowth, wherein the maximum overgrowth was 7 mm. Calvo et al*.* reviewed 27 patients with an average age of 13 years and reported an average overgrowth of 1.6 mm (range, − 4 to 8 mm)^15^. These studies estimated the safety of transphyseal ACL reconstruction in immature bone, so no quantifiable overgrowth was found in their results. Only Zimmerman et al*.* reported 1-cm tibia overgrowth and 1.8-cm femur overgrowth, which were about 2.4% and 3.5% of each bone after transphyseal ACL reconstruction, in a 10-year-old boy^3^. This overgrowth rate of the tibia was similar to our finding. However, we did not make a hole at the physis to avoid direct damage; therefore, the effect of physeal damage was difficult to predict directly.

If physeal damage and interposition with the ligament stimulate overgrowth during ACL reconstruction, overgrowth may not occur in this study because we made the hole beneath the physis. However, we noted overgrowth after creating the metaphyseal hole and interposition with bone wax without physeal violation. Overgrowth has also been reported after all epiphyseal ACL reconstructions^2,16,17^. During all epiphyseal ACL reconstructions, there is no physeal damage, but hyperaemia may occur through epiphyseal drilling. In a previous rabbit study, when the metaphyseal blood supply to the epiphyseal plate was interrupted, the central part of the plate was thickened, and growth was stimulated through hyperaemia^18^. The restoration of suppressed growth was also noted after physeal bar resection or guided growth. Yuan et al*.*^19^ reported an overgrowth of 0.8 cm in the femur and 0.3 cm in the proximal tibia after physeal bar resection. The physeal bar resection is similar to our research protocol in terms of metaphyseal hole creation and bone wax interposition except for physis penetration. In this procedure, there was not a wide periosteal damage, so hyperaemia or decrease tension at the physis would be cause of overgrowth. One review article commented on the rebound phenomenon as an overgrowth after implant removal^20^. There is no physeal damage during eight-plate insertion. The rebound could result from the restoration of physeal function; however, the eight-plate increases the periosteal tension or stability at the physis, and thus, the overgrowth after implant removal may be related to the decrease in periosteal tension or stability. Therefore, in our opinion, the drill hole itself and instability due to the hole may be related to overgrowth through hyperaemia and decreased tension around the physis.

The overgrowth in the metaphyseal hole group (3.17 ± 1.16 mm) was significant compared to that in the sham group, and the amount of overgrowth was comparable to that in the periosteal resection group (2.23 ± 1.52 mm). Periosteal procedures (stripping, division, transection, and resection) are known surgical procedures that have demonstrated the ability to accelerate growth^1,7,8,21–23^. Wilde et al*.*^24^ reported that circumferential periosteal release decreased the leg length inequality by growth stimulation. Limpaphayom et al*.*^1^ performed periosteal stripping and/or division at the shorter limb of 11 children, wherein eight patients achieved limb length equalization. However, this procedure needs a wide incision, and the significant overgrowth was reported only in three cases as 2.3, 2.7, and 4.2 cm of length gain. Furthermore, we still do not know which procedure is the most effective for overgrowth. Periosteal resection created an immediate and sustained acceleration of growth in a lamb study, resulting from axial elongation of the hypertrophic chondrocyte^8^. Halanski et al*.*^7^ compared all reported periosteal procedures in rabbits, including periosteal resection. They concluded that transection of the longitudinally oriented periosteal fibres appears critical to accelerate growth in a rabbit model. The critical procedure at the periosteum is not the width but the transection of the fibre. Additionally, hemicircumferential division of the medial side regularly causes valgus angulation in rabbits^5,6^. During the creation of the metaphyseal hole, the periosteum is partially damaged by the Steinman pin. In our opinion, this small periosteal damage may also be associated with overgrowth.

This study had several limitations. First, although the use of a rabbit model is an economically rational option that is optimal to perform a reproducible operation, it also raises the question of whether these findings apply to clinical practice. The conclusions of this study should be limited to rabbits, until the findings are confirmed in studies of larger animals. Second, we believe that the hyperaemia due to metaphyseal hole creation by cortical breakage, instability due to the large hole, and partial damage to the periosteum during cortical breakage are the causes of overgrowth. However, we could not determine the maximum circumstance for the overgrowth. Further studies dealing with different interposition materials such as a real tendon, different sizes of metaphyseal holes, or a different location of the metaphyseal hole should be conducted. Despite these limitations, it should be noted that this study was the first to evaluate the cause of overgrowth after ACL reconstruction.

Metaphyseal hole creation and interposition with bone wax in rabbits could stimulate overgrowth of the long bone. The amount of overgrowth was comparable to that of the periosteal resection.

## Materials and methods

### Study design and setting

We chose a rabbit model, as the size of the rabbit in a previous study was the smallest among the readily available laboratory mammal that allowed different surgical procedures to be performed in a reproducible manner. We selected 7- to 8-week-old male New Zealand white rabbits. All rabbit experiments were approved by the Committee on the Ethics of Animal Experiments and were performed in accordance with relevant guidelines and regulations. All rabbits were acclimated to the animal care facility for an average of 7 days, approved for use in the study by the facility veterinarian, and underwent the designated surgical procedure. Twenty-one New Zealand white rabbits were obtained from commercial suppliers (male, 2–2.5 kg).

### Study subjects

Periosteal resection (N = 7, periosteal resection group) and metaphyseal hole creation (N = 7, metaphyseal hole group) were performed on the tibiae of skeletally immature rabbits. Seven additional sham controls were included as age-matched controls (sham group). Totally, 21 rabbits were randomly allocated.

### Surgical procedures

**Figure 4:**
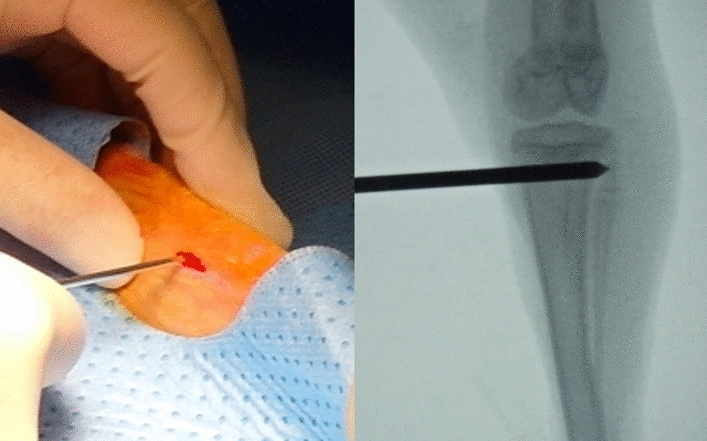
Surgery was performed on the left tibiae of rabbits weighing 2.5–3 kg. A longitudinal medial skin incision was made over the left proximal tibial regions, approximately 1–2 cm in length, in each group. During the creation of the metaphyseal hole, a Steinman pin was used instead of a drill to avoid thermal damage. The vacant space was filled with bone wax.

A longitudinal medial skin incision of approximately 1–2 cm in length was made over the left proximal tibial region for each procedure. The muscles were elevated off the tibia, causing minimal disturbance to the underlying periosteum (sham). In the periosteal resection group, the periosteum was removed below the visible distal medial collateral ligament insertion as a circumferential periosteal strip, 10 mm in width. In the metaphyseal hole group, the hole was made using a Steinman pin at the same level under the image intensifier, and the cancellous bone beneath the physis was removed by curettage (Fig. 4). The vacant space in the metaphysis below the physis was filled with bone wax instead tendon because the tendon is difficult to uptake and could make other damage related to the growth. Tibiae were collected 6 weeks after surgery.

### Outcome measures

Our primary goal was to determine whether the metaphyseal hole creation accelerated growth and increased the bone length. We compared the length of the tibia between the operated and non-operated sides in the metaphyseal hole and the sham control groups. We calculated the overgrowth compared to that of the contralateral limb. We calculated the new bone growth rate using double staining with Calcein Blue. The overgrowth and growth rate in the metaphyseal hole group were compared to those in the sham control group.

Our secondary goal was to compare the effects of metaphyseal hole creation on growth stimulation with periosteal resection. We compared the length of the tibia, overgrowth, and growth rate between the metaphyseal hole and periosteal resection groups.

### Tibial lengths

After harvest, the soft tissues surrounding the tibiae were removed. The bones were then optimally positioned and imaged using a high-resolution REX-650R X-ray system (Listem, Gangwon-do, Korea) and CS-7 software (Konica Minolta, Osaka, Japan). The length was determined by measuring the distance between the tibial eminences proximally to the distal tibial plafond using Image J software (Aspire Software International, Leesburg, VA, USA). The measurement was conducted blindly to minimize the effects of subjective bias.

### Growth rates

For rabbit tibia bone histomorphometry, double labelling, by subcutaneous administration of 25 mg/kg calcein (Sigma-Aldrich Co., St. Louis, MO, USA), 3 and 7 days before sacrifice, was performed. All rabbits were sacrificed 6 weeks after surgery. Both tibiae per rabbit were used for histological analysis. At room temperature, the tibiae were fixed for 1 week in a 3.7% formaldehyde solution. Undecalcified tibia samples were embedded in a plastic resin block, and 50 ± 5 μm sections were produced using a grinding system (EXAKT 400CS, KULZER, Norderstedt, Germany). Fluorescence images of the labelled bone were captured using a Pannoramic 250 Flash III system (Histech, Budapest, Hungary) and analysed using Caseviewer software (3DHistech, Hungary). After bone histomorphometry analysis, the tibiae were extracted from the resin block and decalcified with 0.5 M EDTA for 6 weeks. After paraffin embedding, 5-μm thick sections were cut using a rotary microtome. The sections were sequentially stained using haematoxylin and eosin (H&E) and Masson’s trichrome.

### Statistical analysis

Statistical analyses were performed using SPSS version 23 software (IBM, Armonk, NY, USA). Shapiro–Wilks test was applied to check data distribution. Paired t-test or Wilcoxon signed-rank test was used to compare the tibia length between the operated and non-operated sides. Independent t-test or Mann–Whitney U test was used to compare the overgrowth, height of the growth plate, or growth rate between groups. Statistical significance was set at *P* < 0.05.

### Ethical approval

All rabbit experiments were approved by the Committee on the Ethics of Animal Experiments in Yonsei Biomedical Research Institute, Yonsei University College of Medicine (Permit No. 2020-0102). Animal studies were conducted in line with the ARRIVE guidelines.

## Data Availability

The datasets generated during and/or analysed during the current study are available from the corresponding author on reasonable request.
